# From Human Megakaryocytes to Platelets: Effects of Aspirin on High-Mobility Group Box 1/Receptor for Advanced Glycation End Products Axis

**DOI:** 10.3389/fimmu.2017.01946

**Published:** 2018-01-12

**Authors:** Stefania Mardente, Emanuela Mari, Isabella Massimi, Marco Tafani, Raffaella Guerriero, Ornella Morsilli, Fabio M. Pulcinelli, Marco E. Bianchi, Alessandra Zicari

**Affiliations:** ^1^Department of Experimental Medicine, University of Rome Sapienza, Rome, Italy; ^2^Department of Cellular and Molecular Pathology, IRCCS San Raffaele, Rome, Italy; ^3^Department of Cardiovascular, Dysmetabolic and Aging-Associated Diseases, Istituto Superiore di Sanità, Rome, Italy; ^4^Chromatin Dynamics Unit, San Raffaele University and Scientific Institute, Milan, Italy

**Keywords:** aspirin, platelets, megakaryocytes, high-mobility group box 1, receptor for advanced glycation end products

## Abstract

Platelets (PLTs) are the major source of high-mobility group box 1 (HMGB1), a protein that is involved in sterile inflammation of blood vessels and thrombosis. Megakaryocytes (MKs) synthesize HMGB1 and transfer both protein and mRNA into PLTs and PLT-derived microvesicles (MV). Free HMGB1 found in supernatants of *in vitro* differentiated MKs and in a megakaryoblastic cell line (DAMI cells). Aspirin “*in vivo*” and “*in vitro*” not only reduces HMGB1 and receptor for advanced glycation end products expression on MKs and PLTs but also drives the movement of HMGB1 from MKs into PLTs and PLT-derived MV. These findings suggest that consumption of low doses of aspirin reduces the risk of atherosclerosis complications as well as reducing PLT aggregation by the inhibition of COX-1.

## Introduction

Platelets (PLTs) are involved in hemostasis, thrombosis, and immunity. Apart from PLT plug formation and releasing coagulation factors, PLTs are important for vessel remodeling and deposition of constituents that are active in extravascular matrix. Inflammation and coagulation are connected and regulated by common pathways and PLTs provide a link between coagulation and inflammation (1, 2).

Platelets arise from progenitor megakaryocytes (MKs) that provide them with peptides that are active in inflammation and coagulation and with mRNAs that are translated upon their activation.

One of the molecules investigated for its role in both coagulation and inflammation is high-mobility group box 1 (HMGB1) (3, 4), which is known to be produced in MKs and PLTs (5), as in most other cells. HMGB1 is a damage-associated molecular pattern that when released by stressed cells starts inflammation. Levels of HMGB1 increase in plasma and serum of patients with inflammatory diseases associated with sepsis or thrombosis. Studies performed in transgenic mice with ablation of PLT-specific HMGB1 (*PLT-specific HMGB1-knockout*) and in an *in vivo* model of thrombosis (FeCl_3_-induced), demonstrated that PLT-derived HMGB1 promotes PLT aggregation and small vessel thrombosis (6).

High-mobility group box 1 is a nuclear protein that is also active in the cytoplasm and in the extracellular space. It has recently been shown that when HMGB1 relocalizes from the nucleus to the cytoplasm, it is acetylated and mostly reduced, while when it is secreted by immune cells is acetylated. After release in the extracellular medium, cysteines C23 and C45 of HMGB1 can form a disulfide bond (7, 8). Disulfide HMGB1 is recognized by surface and intracellular TLRs. Further oxidation of cysteines to sulphonates inactivates HMGB1. However, its most interesting binding partner is receptor for advanced glycation end products (RAGE), which is bound by different ligands that trigger inflammatory states. RAGE is a type I transmembrane protein and a member of the immunoglobulin superfamily, it is increasingly expressed when potential ligands such as HMGB1 or inflammatory mediators are expressed, e.g., in cardiovascular disease, diabetes, and cancer (9–11). RAGE-dependent mechanisms have also been hypothesized to mediate PLT activation (12).

Furthermore, it has been demonstrated that PLT activation increases RAGE surface expression and that RAGE binds HMGB1 released in thrombi by endothelial cells and leukocytes. Thus, HMGB1–RAGE interaction is central in the pathogenesis of atherothrombosis. This suggests that the HMGB1–RAGE pathway should be targeted in prevention and therapy of vascular damage in thrombosis disease (13, 14).

Aspirin is known to permanently inhibit COX-1 in mature PLTs and in MKs. For this reason, low dosages of aspirin (100 mg) are administered daily in order to prevent thrombosis. The fact that 10–15% of PLTs are renewed daily but only 3 or 4% of circulating PLTs show COX-1 function suggests that low doses of aspirin administered once a day function on PLT progenitors (15). As well as acting on COX-1, aspirin has been shown to influence megakaryocytic gene expression, by activating nuclear factor PPARα that consequently upregulates multidrug resistance protein 4 in human PLTs (15). Other authors have demonstrated that aspirin induces over-expression of GP3A (16) and a complex of genes named “aspirin response signature” (17).

More recently, aspirin (or its de-acetylated derivative, salicylic acid) has been shown to bind HMGB1 and inhibit its activities (18). Extracellular HMGB1 induces the transcription of Ptgs2, the gene coding for COX-2, and salicylic acids inhibits such induction. Salicylic acid has been hypothesized to be anti-tumorigenic in mesothelioma and in colon cancer (19).

A modulation of HMGB1 expression induced by aspirin could represent another mechanism contributing to the anti-PLT effects of the drug.

The aim of this study is to investigate whether HMGB1 has a role in the biogenesis of PLTs and whether it could be influenced by aspirin.

Aspirin effects on HMGB1 expression and release were studied in a human megakaryoblastic cell line (DAMI cells), in PLTs obtained from healthy volunteers (HVs) and from patients with high-thrombotic risk.

## Materials and Methods

### Cell Cultures

DAMI cells were maintained in RPMI 1640 medium supplemented with 10% fetal calf serum, penicillin G sodium (100 U/ml) and streptomycin sulfate (0.1 mg/ml), in a humidified atmosphere (5% CO_2_, 37°C). DAMI cell differentiation was induced with 1 mM phorbol myristate acetate and 10 ng/ml thrombopoietin (TPO) for 7 days, and cells were cultured for four more days without stimulation (20). Where required, at day 6 of differentiation, cells were treated with 50 µM aspirin (Sigma) for 4 days.

### Human Hematopoietic Progenitor Cell (HPC) Purification

Adult peripheral blood (PB) was obtained from 20- to 40-year-old male donors after written informed consent. Low-density mononuclear cells (in average 0.1%) were isolated by Ficoll–Hypaque (Lympholyte CL5020, Cederlane Lab., Canada) density gradient (1.077 g/ml) centrifugation at 600 *g* for 30 min, RT. CD34^+^ cells were purified by using the MiniMACS isolation system (Milteny, Bergisch, Gladbach, Germany) according to the manufacturer’s instructions. Purified cells were more than 90% CD34^+^ (as evaluated by cytofluorimeter, Epics Profile) and were cultured in serum-free FCS unilineage MK liquid culture (20), with addition of 100 ng/ml TPO alone or in combination with 50 µM aspirin. Aspirin treatment started from day 6 of culture and continued for the following 4 days. Cultures were maintained for 14 days in a humidified atmosphere (5% CO_2_, 37°C). MKs were collected, counted, and analyzed for viability and morphology at different days of differentiation (7, 9, and 14). PLTs, obtained at the end of the culture, were isolated from supernatants by centrifugation at 800 *g* for 10 min at room temperature. Supernatants were centrifuged a second time at 14,000 *g* for 10 min in order to remove debris and were kept at −20°C before ELISA tests.

### PLT Preparation and Isolation

Donors were divided into four groups as summarized in Table 1. 10 HVs (group 1), 10 HVs who were administered 300 mg/day aspirin (group 2), 10 high-cardiovascular risk patients (group 3), and 10 high-cardiovascular risk patients who were under therapy with aspirin (100 mg *per os* for at least 3 months) (group 4). This study was carried out in accordance with the recommendations and approval of the ethical committee of Policlinico Umberto I-University of Rome Sapienza with written informed consent from all subjects. All subjects gave written informed consent in accordance with the Declaration of Helsinki.

**Table 1:** Description of donors used in the study.

Subjects	Group 1, healthy volunteers (HVs)	Group 2, HVs (ASA 300 mg/day *per os*)	Group 3, high-risk thrombosis patients	Group 4, high-risk thrombosis patients (ASA 100 mg/day/*per os*)
Total number	10	10	10	10
Male/female	6/4	6/4	7/3	7/3
Age range	25–55	25–55	58–75	58–75

Platelets, microvesicles (MV) extracted from plasma, serum, and platelet-free plasma, were obtained from each donor. Blood samples were centrifuged at 200 *g* for 15 min and platelet rich plasma (PRP) was collected. ACD (39 mM citric acid, 75 mM sodium citrate, and 135 mM dextrose) was added to PRP, in order to avoid PLT aggregation. This was then centrifuged at 1,000 *g* for 10 min to remove plasma. Pellets containing PLTs were resuspended in ACD buffer with EDTA (5 mM) and then filtered through a 5-µm filter to remove leukocyte contaminants (21, 22).

Supernatants (PPP) obtained after centrifugation of PRP were centrifuged again at 20,000 *g* for 10 min to obtain MV. Pellets were used for western blot. Supernatants were used for ELISA tests.

### Flow Cytometry Analysis of Cell Surface Antigens

The phenotype of differentiating MKs and PLTs was analyzed using FITC or PE conjugated monoclonal antibodies anti-CD34 (Becton Dickinson) and anti-CD61 (BioLegend). Cells were incubated with antibody (diluted 1:100 for 15 min), washed with PBS, and analyzed in a cytofluorimeter (Epics XL–MCL Coulter).

### mRNA Extraction and RT-PCR

Total RNA from human cell lines, MKs, and human PLTs was extracted using TRizol reagent (Invitrogen, San Diego, CA, USA). For mRNA detection, 1 µg of total RNA was transcribed using the GeneAmp Gold RNA PCR reagent kit pAW109 (Applied Biosystems, Warrington, UK). mRNA expression was analyzed with Q-RT-PCR using TaqMan Master Mix and TaqMan assay reagents (Applied Biosystems). PCR conditions were as follows: 50°C for 2 min and 95°C for 10 min, followed by 40 cycles of 95°C for 15 s and 60°C for 1 min. Amplification was carried out in triplicate. β-actin mRNA was used for normalization. A negative control (no cDNA) was used to confirm the absence of amplification.

### Protein Extraction and Western Blot

Cell pellets were resuspended in lysis buffer [RIPA buffer: 10 mM Tris–HCl (pH 7.6), 160 mM NaCl, 1 mM EGTA, 1% deoxycholic acid, 1%Triton, and 0.1% SDS] with protease inhibitors, incubated on ice for 30 min and then centrifuged at 12,000 *g* for 30 min; supernatants were collected. Whole cell lysates were heat denatured for 5 min and separated on 10% SDS-PAGE gels, run on ice at 40 V (for the stacking gel) and 80 V (for the running gel). Proteins were transferred onto a previously activated PVDF membrane (Bio-Rad, Hercules). Membranes were then placed in TBS-T and 5% albumin for 1 h and probed overnight with the specific antibody at 4°C. At the end of incubation, membranes were washed and incubated with anti-mouse IgG peroxidase conjugated secondary antibody (1:10,000) for 1 h at room temperature. Membranes were stripped and incubated with anti-actin monoclonal antibody (Sigma) as a loading control. Signal was detected by autoradiography (Kodak Biomax) using the chemiluminescent peroxidase substrate kit (Sigma) and then quantified by densitometry (Bio-Rad).

### Immunofluorescence and Confocal Microscopy

For immunofluorescence, cells were spotted on a glass slide, fixed with 4% paraformaldehyde in PBS for 30 min, washed with 0.1 M glycine for 20 min, and permeabilized in 0.1% Triton X-100 for an additional 5 min. Nuclei of MKs were stained with Sytox green Nucleic Acid stain (Invitrogen), according to manufacturer’s instructions. Primary antibodies (anti-RAGE or anti-HMGB1) were diluted according to manufacturer’s instructions and added on spots for 45 min, then slides were washed and secondary antibodies (PE- or FITC-labeled anti-IgG) were added for 30 min. At the end, slides were washed with PBS, mounted with coverslips, and kept at 4°C until imaging with confocal microscopy (Zeiss LSM-510).

### Statistics

All statistical analysis was carried out using KaleidaGraph version 4.5.1 (Synergy Software Inc., Reading, PA, USA). Data are expressed as means ± SD. The differences between differently treated cell populations were analyzed using Student’s *t*-test. Differences among groups were determined by using one-way ANOVA and Dunnet *post hoc* test. *p* < 0.05 was considered to indicate a statistically significant difference.

## Results

### HMGB1 Expression Is Modulated by Aspirin in DAMI Cells

**Figure 1:**
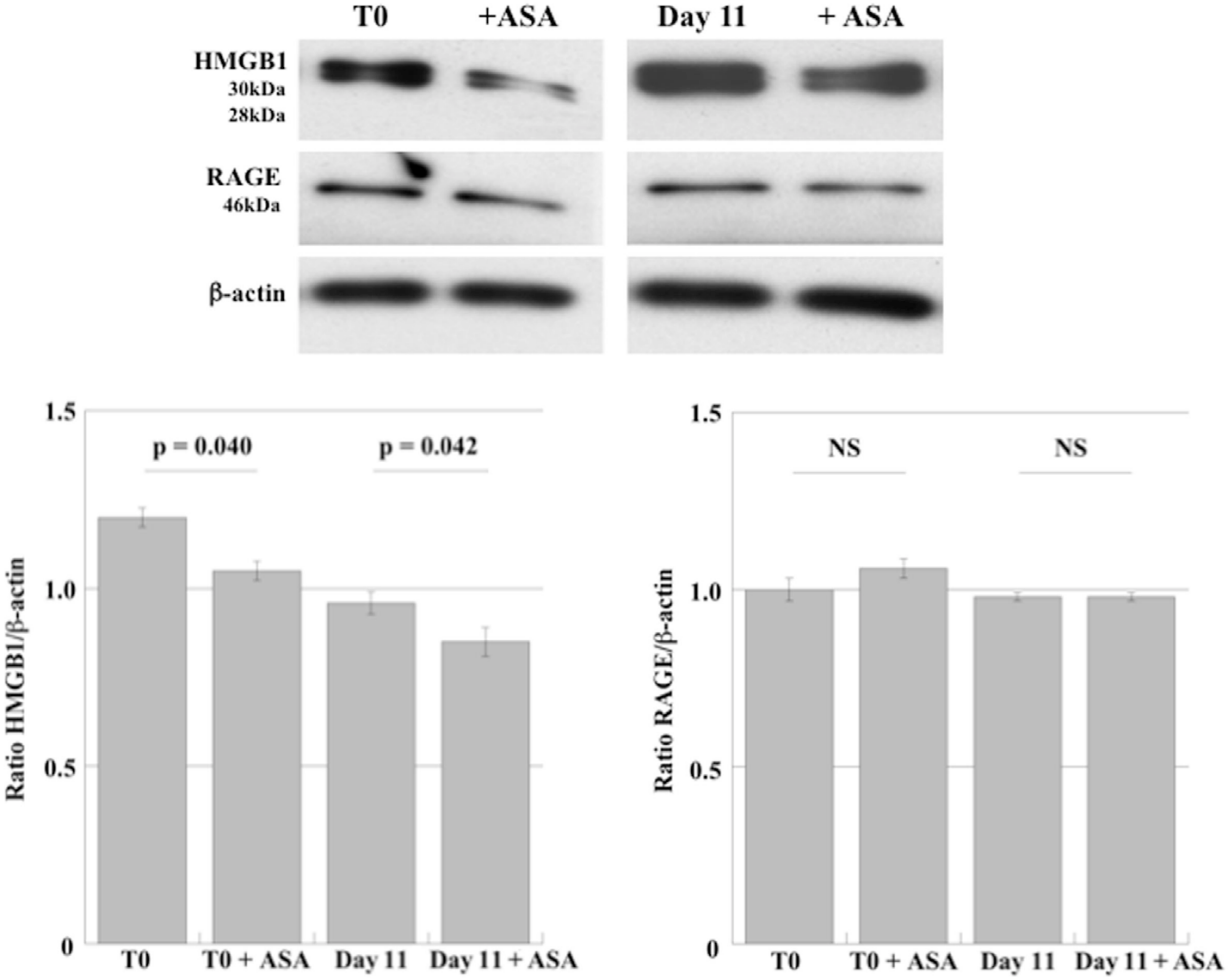
HMGB1 and RAGE expression in undifferentiated DAMI cells (T0) and in DAMI cells at day 11 of differentiation treated or not with aspirin (50 µM). The expression level of HMGB1 (28–30 kDa) and RAGE (43 kDa) was detected by western blotting in whole cell lysates. Results are representative of three different experiments. Statistical differences were evaluated by Student’s *t*-test.

High-mobility group box 1 and RAGE expression were first investigated in DAMI cells during their differentiation to PLTs. In order to evaluate whether aspirin affect HMGB1 and RAGE expression, we also treated undifferentiated and differentiated DAMI cells with aspirin. As shown in Figure 1, DAMI cells express HMGB1 and treatment with 50 µM aspirin slightly reduced its expression in both undifferentiated (T0) and differentiated cells (day 11).

Receptor for advanced glycation end products was constantly expressed in DAMI cells at the different stages of differentiation (Figure 1) and treatment with aspirin did not induce any significant effect.

**Figure 2:**
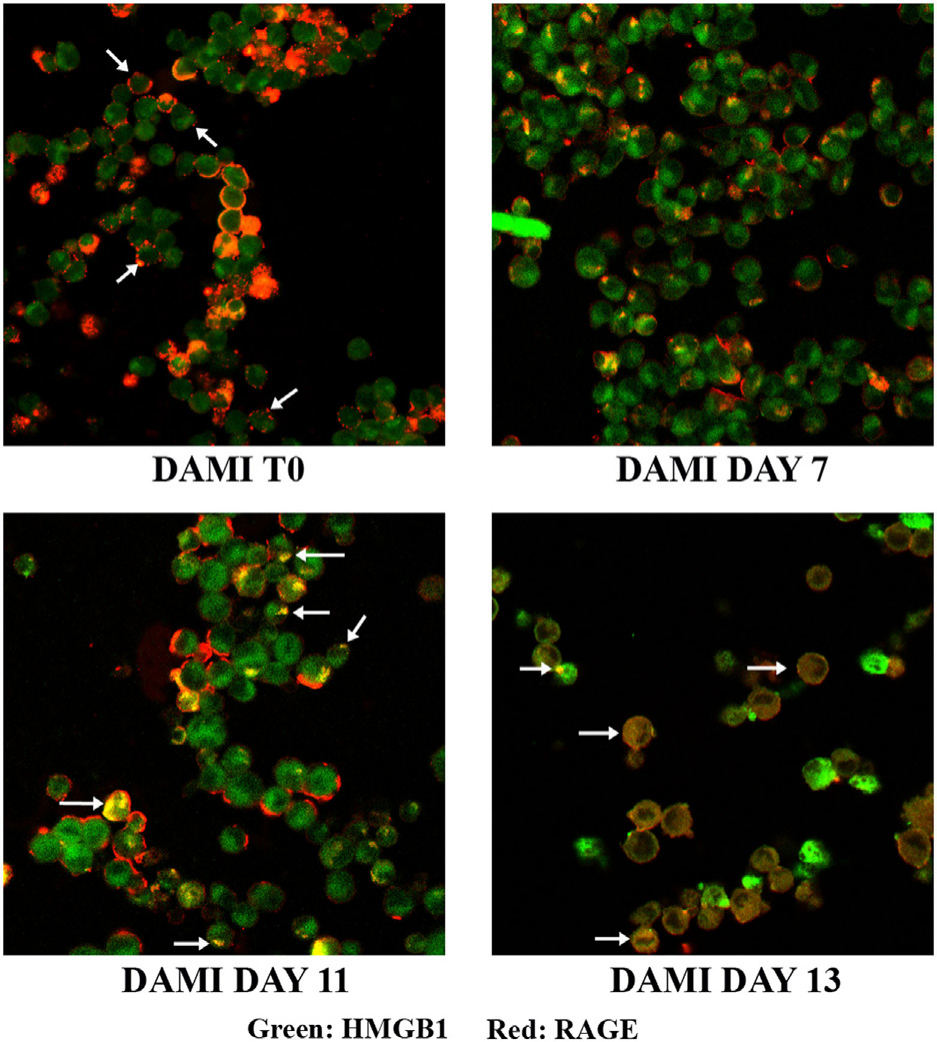
HMGB1 and RAGE localization in DAMI cells: from undifferentiated (T0) to fully differentiated. HMGB1 (green) and RAGE (red) localization were detected by confocal analysis. Upper left: untreated DAMI cells showing patchy distribution of RAGE on the plasmamembrane (arrows) and nuclear and cytosolic distribution of HMGB1. At day 11, DAMI cells show patched co-localization of HMGB1 and RAGE as indicated by the arrows. At day 13, cells show diffuse co-localization of HMGB1 and RAGE (white arrows).

**Figure 3:**
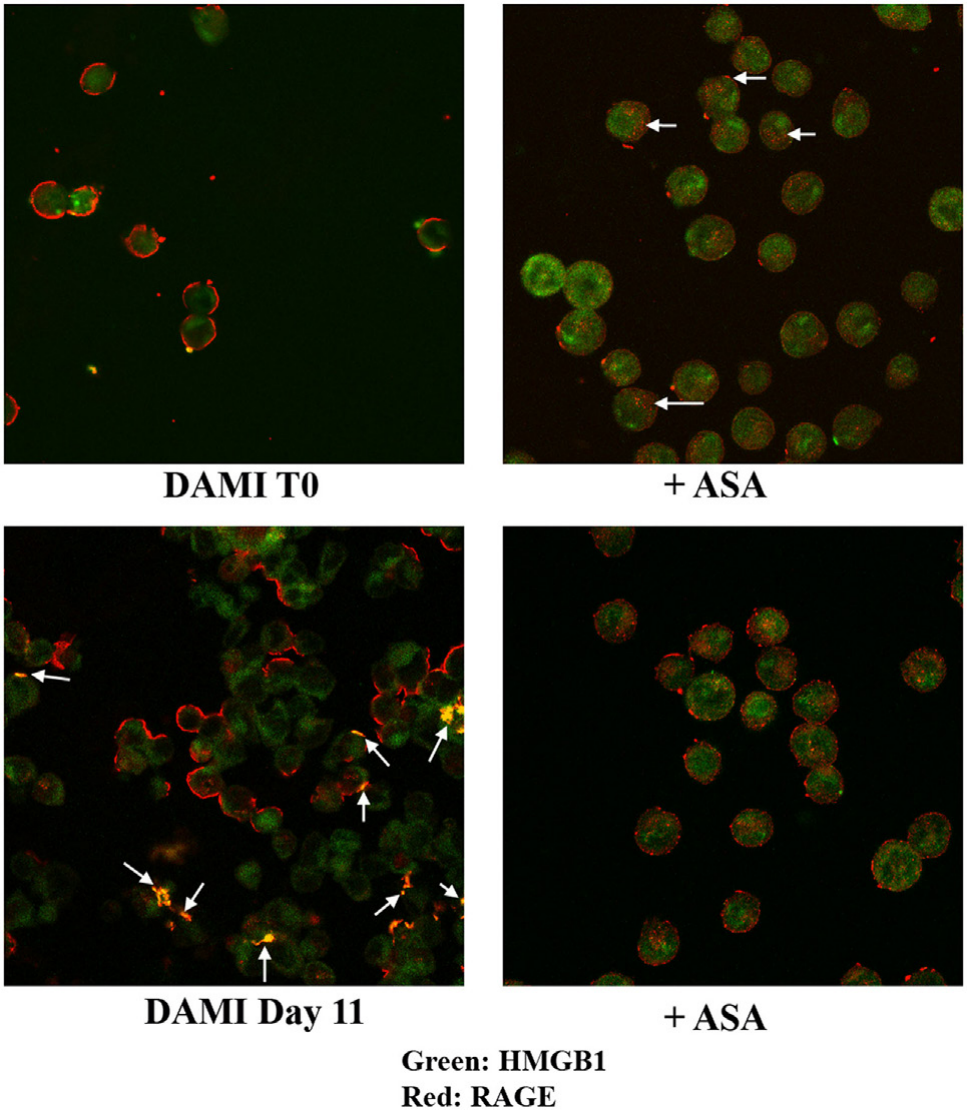
HMGB1 and RAGE localization in undifferentiated (T0) or differentiated DAMI cell treated or not with aspirin. HMGB1 (green) and RAGE (red) localization were detected by confocal analysis. Upper left: untreated proliferating DAMI cells showing a patchy distribution of RAGE (red) on the plasmamembrane and nuclear localization of HMGB1 (green). Upper right: proliferating DAMI cells treated with 50 µM ASA. Note the redistribution of RAGE, which accumulates in the cytosol as pointed by the arrows. Lower left: day 11, DAMI cells showing some co-localization of HMGB1 and RAGE on the plasmamembrane (arrows). Lower right: DAMI cells treated with 50 µM ASA do not show co-localization of HMGB1 and RAGE, and have a diffused distribution of RAGE.

Cellular localization of HMGB1 and RAGE was studied using confocal microscopy. Undifferentiated cells show nuclear and cytosolic localization of HMGB1 whereas RAGE is localized in the plasmamembrane (Figure 2, upper left). Interestingly a large number of undifferentiated DAMI cells show a patchy distribution of RAGE on the plasmamembrane, as shown by the arrows in Figure 2. In differentiating DAMI cells, at day 7 localization of HMGB1 and RAGE was similar to that in T0 cells, and RAGE was distinctly patchy (Figure 2, upper right). However, as differentiation proceeded, at day 11 there was an increased plasmamembrane co-localization of HMGB1 and RAGE (yellow spots, see the arrows in Figure 2, lower left). Finally, after 13 days of differentiation, there was a clear redistribution and co-localization of HMGB1 and RAGE which was both diffused in the cytoplasm and patched on the plasmamembrane (see the arrows in Figure 2, lower right). When undifferentiated DAMI cells were treated with ASA, we observed a redistribution of both RAGE and HMGB1; RAGE was more intracellular (see the arrows in Figure 3, upper right). Differentiated DAMI cells under ASA treatment at day 11 maintained a widespread distribution of both HMGB1 and RAGE. Moreover, we did not observe co-localization of HMGB1 and RAGE as in untreated cells (see Figure 3, lower right).

### HMGB1 Expression Is Modulated by Aspirin in Human MKs

**Figure 4:**
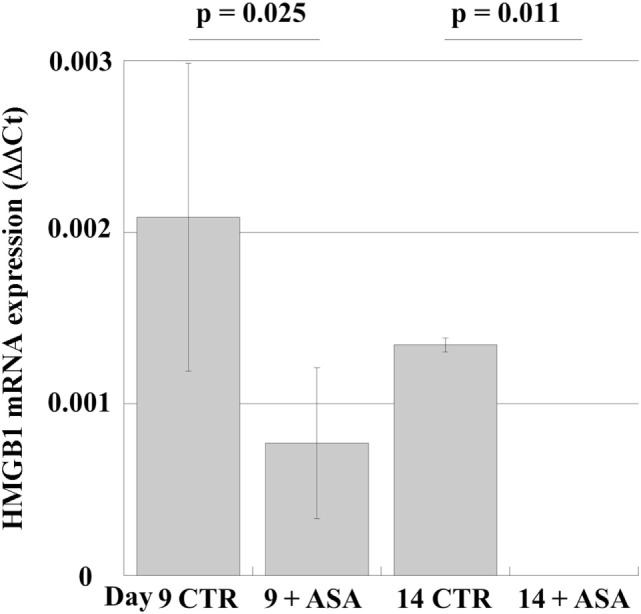
mRNA expression in cultures of megakaryocytes, obtained from peripheral-blood precursors (HPCs) and induced to differentiate in multiwell plates. At day 6 of culture 50 µM aspirin (+ASA) was added for the following 4 days. RT-PCR was performed at days 9 and 14. Statistical differences were evaluated by Student’s *t*-test.

In order to examine whether aspirin could modulate HMGB1 expression in human MKs, we studied the effects of aspirin in peripheral-blood progenitor cell cultures during maturation along the MK lineage (19). Aspirin (50 µM) was added at day 6 to differentiating MKs (when most cells are precursors) and incubation was continued for 4 days. The results (Figure 4) show that mRNA for HMGB1 is expressed at the different stages of differentiation (days 9 and 14). Treatment with ASA decreases mRNA expression until, on day 14 of differentiation, it became undetectable.

**Figure 5:**
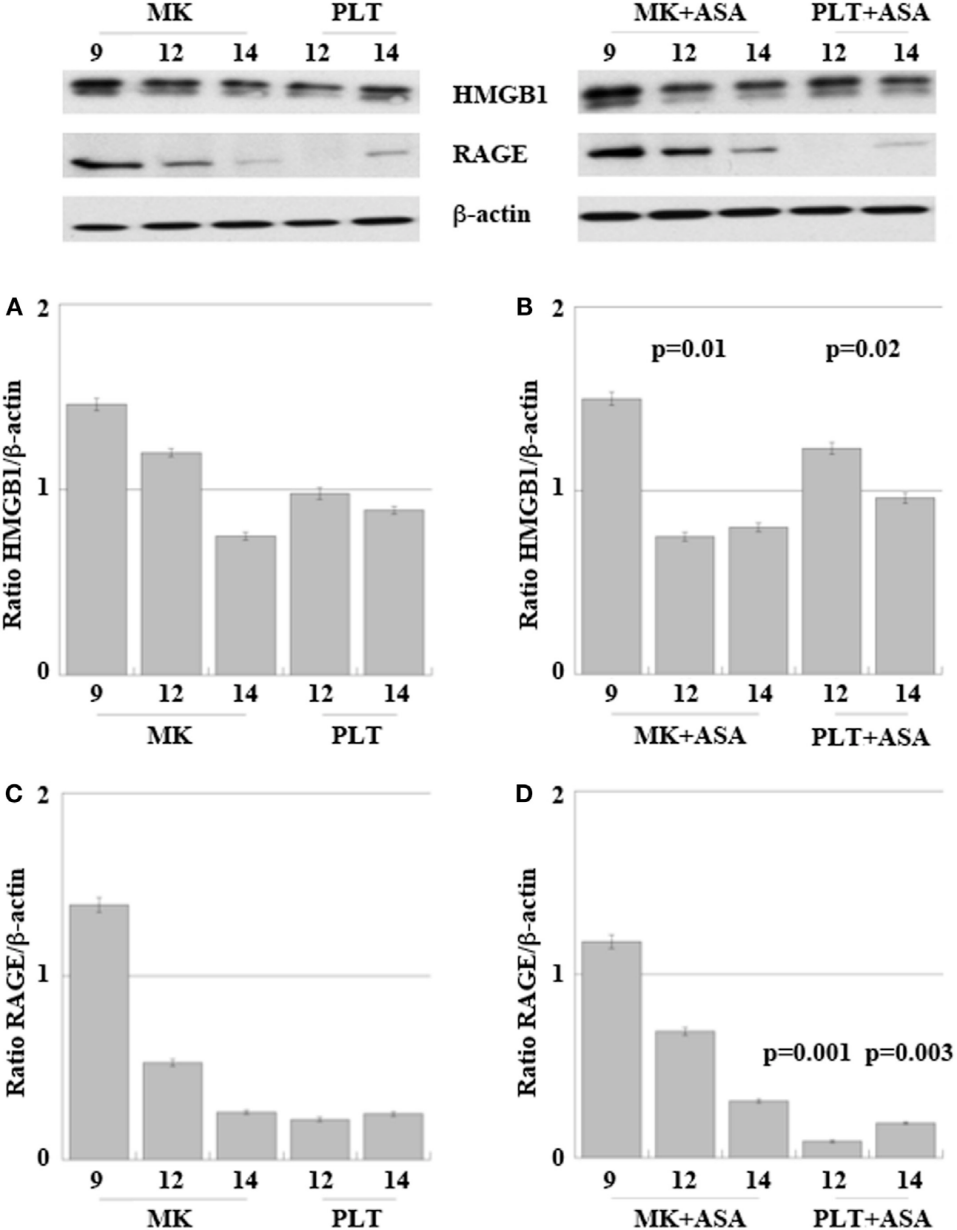
High-mobility group box 1 (HMGB1) **(A,B)** and receptor for advanced glycation end products (RAGE) **(C,D)** expression in megakaryocytes (MKs) obtained from peripheral HPCs and induced to differentiate as described in Section “Materials and Methods.” Platelets (PLT) were obtained at days 12 and 14 of differentiation **(A–D)**. 50 µM aspirin was added to cultures at day 6, for 4 days **(B,D)**. Results are representative of three different experiments. Statistical differences were evaluated by one-way ANOVA test and Dunnet *post hoc* analysis in MKs + ASA versus MKs and PLT + ASA versus PLT (*p*-values are indicated where statistically significant).

Figure 5 shows expression of HMGB1 and RAGE proteins in differentiating MKs collected at days 9, 12, and 14 and in their derived PLTs, treated or not with aspirin.

At day 9 of *in vitro* maturation, MKs begin to produce PLTs CD61+ (see the cytofluorimetric analysis in Figure S1 in Supplementary Material). Interestingly (Figure 5A), HMGB1 is decreasingly expressed in MKs during days 9, 12, and 14 of maturation, while at the same time it starts appearing in the newly formed PLTs. This means that HMGB1 is distributed into the future PLTs by the progenitors. The more cells progress into the maturation and get close to the production of PLTs, the more HMGB1 is moved from the cell nucleus to the periphery of the cells (shown in Figure S2 in Supplementary Material) and into their derived PLTs. Treatment with aspirin decreases the amount of HMGB1 in MKs, while it slightly increases it in the PLTs (Figures 5A,B). This slight increase of HMGB1 expression in the new PLTs is not followed by a higher amount of free protein released in supernatants as detected by ELISA and shown in Table 2. RAGE expression followed the same trend of expression as HMGB1 in MKs while it markedly decreased in PLTs obtained from cultures treated with aspirin for 12 and 14 days (Figures 5C,D).

**Table 2:** Content of high-mobility group box 1 (ng/ml) in supernatants from megakaryocytes (MKs) detected by ELISA.

Day of maturation and treatment	Supernatants
MK day 9	18.25 ± 0.3
MK day 9 + ASA	17.45 ± 0.2
MK day 12	19.45 ± 0.2
MK day 12 + ASA	18.02 ± 0.3
MK day 14	22.19 ± 0.2
MK day 14 + ASA	20.13 ± 0.3

*Each value is the mean ± SD ng/ml of three determinations in three different experiments performed on different days. Supernatants were obtained from MKs from HPC at day 9 or 12 of maturation, treated or not with aspirin (see Materials and Methods)*.

### HMGB1 Expression in PLTs Obtained from High-Atherothrombotic Risk Patients

To investigate whether aspirin could regulate HMGB1 expression and secretion *in vivo*, we studied the content of HMGB1 in PLTs, MV, plasma, and in sera from high-risk thrombosis patients treated or not with aspirin (groups 3 and 4).

**Figure 6:**
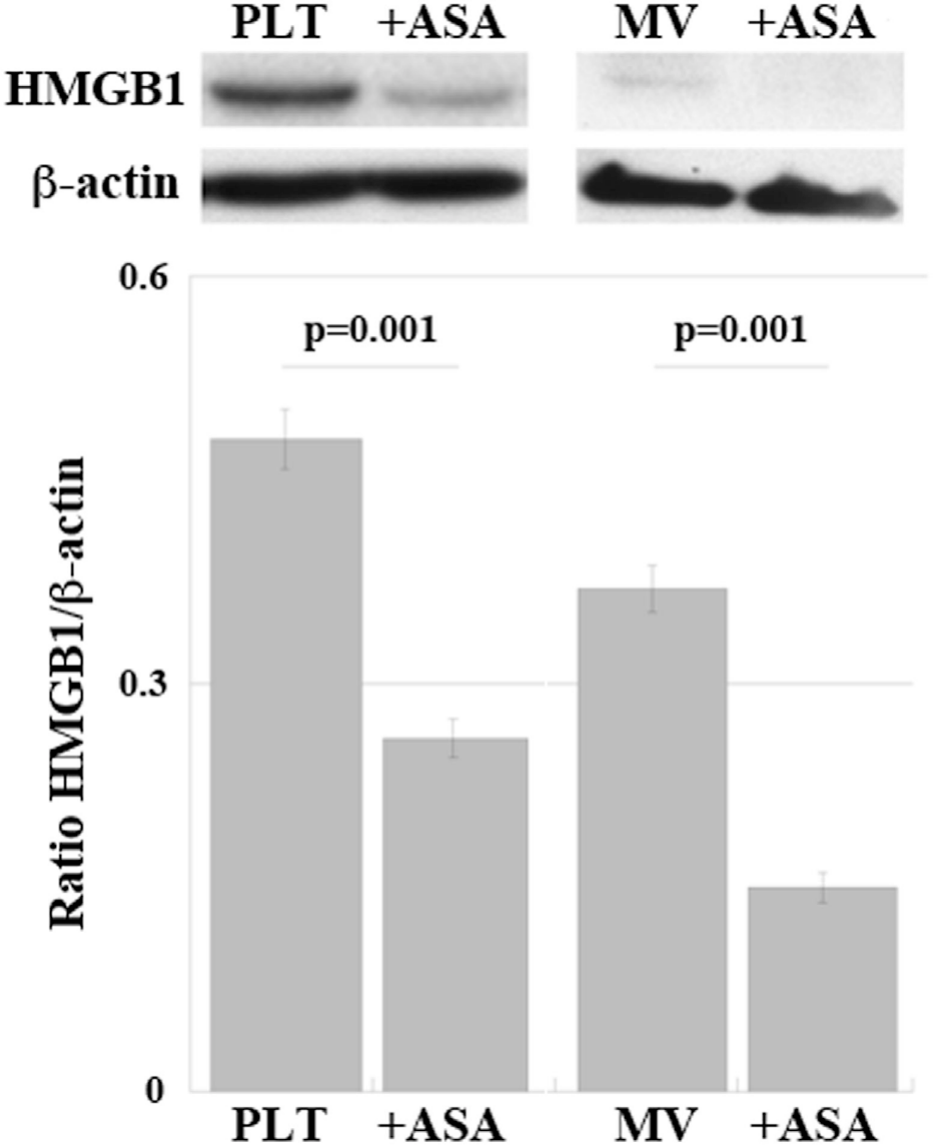
Expression of high-mobility group box 1 (HMGB1) in platelets (PLT) and PLT-derived microvesicles (MV) obtained from high-atherothrombotic risk patients treated or not with ASA (100 mg/die *per os*). Representative western blot. Statistical differences were evaluated by Student’s *t*-test.

Representative western blots regarding expression of HMGB1 in PLTs and in MV are shown in Figure 6. There is a decrease of expression of HMGB1 in both PLTs and MV from patients under aspirin treatment (100 mg/day for at least 3 months). Table 3 shows the concentration of soluble HMGB1 in plasma and sera from the same patients. There is a correspondence between western blots of PLTs, MV, and free protein measured in plasma and sera from the same subjects.

**Table 3:** Content of high-mobility group box 1 (HMGB1) (ng/ml) detected by ELISA.

	HMGB1 nanograms per milliliter (detected by ELISA)
Plasma from high-risk thrombosis patients (group 3)	0.32 ± 0.2
Serum (group 3)	1.27 ± 0.2
Plasma from HRP in treatment with ASA (group 4)	0.18 ± 0.3
Serum (group 4)	0.41 ± 0.4

*Each value is the mean ± SD ng/ml of three different determinations performed in plasma and serum obtained from patients described in Table 1*.

Interestingly, as shown in Table 3, there is a higher expression of HMGB1 in sera than in plasma due to the activation of PLTs during clot formation, with release of HMGB1.

### HMGB1 mRNA Expression in PLTs Obtained from HVs

**Figure 7:**
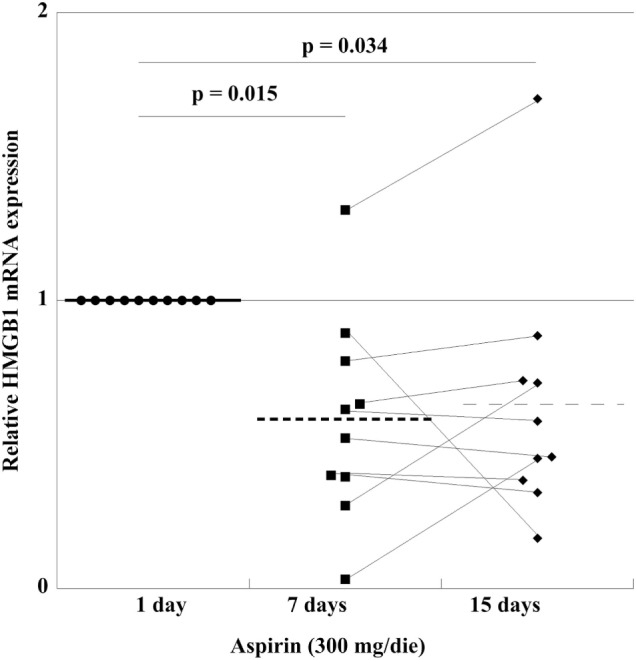
High-mobility group box 1 (HMGB1) mRNA expression in platelets from 10 volunteer subjects, before treatment (healthy volunteers CTRL), and after aspirin treatment (300 mg/die) for 7 and 15 days. Statistical differences were evaluated by one-way ANOVA and Dunnet *post hoc* analysis.

In order to find out whether *in vivo* treatment with aspirin had an effect on the mRNA content of HMGB1 in PLTs, we studied the mRNA expression in a group of HVs before and after 300 mg/day aspirin for up to 15 days. As shown in Figure 7, there is a decrease of expression at day 7, which is followed by a small increase when treatment is prolonged for 15 days, well under the levels before aspirin administration. This means that *in vivo* treatment with aspirin regulates HMGB1-expression and disposition into PLTs at the megakaryocytic level.

## Discussion

In the present study, we investigated the expression of HMGB1 and RAGE and their modulation by aspirin in MKs, obtained from PB HPC and in a megakaryoblastic cell line, DAMI, which has proved to be a good model for the study of megakaryocytic differentiation (23). In particular, we have shown that aspirin, during *in vitro* differentiation of DAMI cells directs the movement of HMGB1 from nucleus to cytoplasm: in undifferentiated cells HMGB1 is mostly in the nucleus and, during differentiation it is in the cytoplasm, gets closer to the plasma membrane and we suggest is expressed in the buds that would probably give rise to new PLTs. Yamashita et al. (24) analyzed thrombi from patients with coronary thrombosis and found that HMGB1 was closely localized with PLTs. They also suggest that increased serum levels of HMGB1 in patients with type 2 diabetes mellitus is due to impaired release of HMGB1 from leukocytes. We are suggesting here that PLTs are provided with HMGB1 by their progenitors and that PLTs are the major source of HMGB1 in thrombi because they are also provided by MKs with mRNA HMGB1. PLTs have a dual role: initiate blood clotting and start inflammation. HMGB1 is one of the molecules that links both PLT functions. Since the major inhibitor of PLT functions is aspirin, we are suggesting here that aspirin is able to modulate HMGB1 expression on MKs and in their derived PLTs.

Aspirin pharmacokinetics and pharmacodynamics *in vivo* depend on gastro intestinal absorption and on the life span of PLTs and MKs. A recent model on anti-PLT pharmacodynamics of low-dose aspirin in humans (18) has suggested that a faster recovery of COX-1 activity in PLTs could be obtained by shortening the interval of administration and not by increasing the dose. In the present study, therapeutic doses of aspirin were administered to patients (100 mg/die) and HVs (300 mg/die), so the range of plasma levels of aspirin was between 15 and 50 µM.

Aspirin induces similar effects in human MKs obtained from HPC during their “*in vitro*” maturation: HMGB1 mRNA decreased from day 7 of differentiation in ASA-treated cells until it became undetectable at day 14. It is interesting to note that, as with DAMI cells during differentiation, cellular content of HMGB1 protein also decreased. This suggests, that HMGB1 is synthesized at the initial stages of cell differentiation and that most of it is then distributed into PLTs and then again from PLTs into PLT-derived MV. Recent studies (25) demonstrated that PLT-derived exosomes contain HMGB1 among α-granule markers and more interestingly, this content is decreased by consumption of low-dose aspirin daily for 1 week.

Having shown that aspirin plays a role in the expression and transport of HMGB1 from MKs into PLTs, we investigated the effects of oral treatment with aspirin in young, HVs, and older cardiovascular risk patients and demonstrated that aspirin is active MK gene expression and that MKs provided PLTs with mRNA HMGB1 as well as protein. When PLTs are activated, they release HMGB1 and are also able to produce it from mRNA, meaning that their potential to produce HMGB1 during activation is more powerful than it would be if they only expressed the protein.

Moreover, aspirin is able to reduce the amount of mRNA, inherited from progenitors, in HV PLTs and therefore a minor amount of protein available in thrombi.

The fact that HMGB1 content in serum, PLTs, and derived MV increases with age (25) needs careful consideration since the powerful procoagulant function exerted by HMGB1 may contribute to pathogenesis of thrombosis (26). In the light of recent studies carried out in mice, HMGB1 not only induces thrombosis and inflammation but also tissue repair and regeneration (27). Moreover, it contributes to formation of neutrophil extracellular traps, which have been shown to play a role in formation and growth of peripheral thrombi (28). Furthermore, HMGB1 contained in PLT-derived MV is released during PLT activation in patients with systemic sclerosis (29). We confirm here that HMGB1 content in PLTs and in their derived MV in high-risk thrombosis patients is increased in comparison with PLTs from HVs of a younger age group and that treatment with aspirin reduces HMGB1 expression in PLTs and in PLT-derived MV. The amount of free HMGB1 released in plasma is also decreased by treatment with aspirin. It has been shown (30) that aspirin consumption reduces the *in vivo* plasma levels of many PLT constituents that play active roles in thrombosis, therefore preventing thrombosis. Another interesting observation the present study is that aspirin also reduces the expression of RAGE in DAMI cells, MK, and their derived PLTs.

Receptor for advanced glycation end products is a multiligand receptor expressed in various tissues and in a variety of cells, including endothelial cells and PLTs (31). The interaction of RAGE with HMGB1 or with its other ligands, such as S100 proteins and β-amyloid, induces NF-κB activation, release of relevant pro-inflammatory cytokines and progression of atherosclerotic plaques. Moreover, RAGE could exert its pro-thrombotic action by interacting with ICAM-1 expressed on endothelial cells, causing the release of chemokines that contribute to pro-thrombotic events (31). The study presented here concerned RAGE when ligated to HMGB1 and as we are not aware of any direct interaction of aspirin with RAGE, we suggest that the entire HMGB1−RAGE axis is regulated by aspirin at the central level in MKs. We also suggest that the decreased expression of both HMGB1 and RAGE, could be of great significance in the reduction of cardiovascular complications due to their combined action.

## Author Contributions

EM designed the work and performed the experiments was responsible for data presentation and figures. SM (corresponding author) designed the work, interpreted the data, directed the experiments, and wrote the paper. RG and OM performed human hemopoietic progenitor cell (HPC) purification and cellular differentiation, critically revised the manuscript. MT performed confocal analysis, interpreted the data, and critically revised the manuscript. IM performed the mRNA experiments and interpreted the data was responsible for Figures 4 and 7. FP designed the work, selected the patients and donors and was responsible for collection of samples, critically revised the manuscript, and wrote the paper. MB drafted the work, critically revised the manuscript, and wrote the paper. AZ designed the work and wrote the paper.

## Conflict of Interest Statement

The authors declare that the research was conducted in the absence of any commercial or financial relationships that could be construed as a potential conflict of interest. The reviewer MC and handling editor declared their shared affiliation.
