# Supplementary material for: From Human Megakaryocytes to Platelets: Effects of Aspirin on High-Mobility Group Box 1/Receptor for Advanced Glycation End Products Axis
Source: Front Immunol. 2018 Jan 12;8:1946. doi: 10.3389/fimmu.2017.01946 (PMC5770369; doi:10.3389/fimmu.2017.01946)
Supplement: Supplementary file 1 [file Image_1.PDF]

Supplementary Figure 1

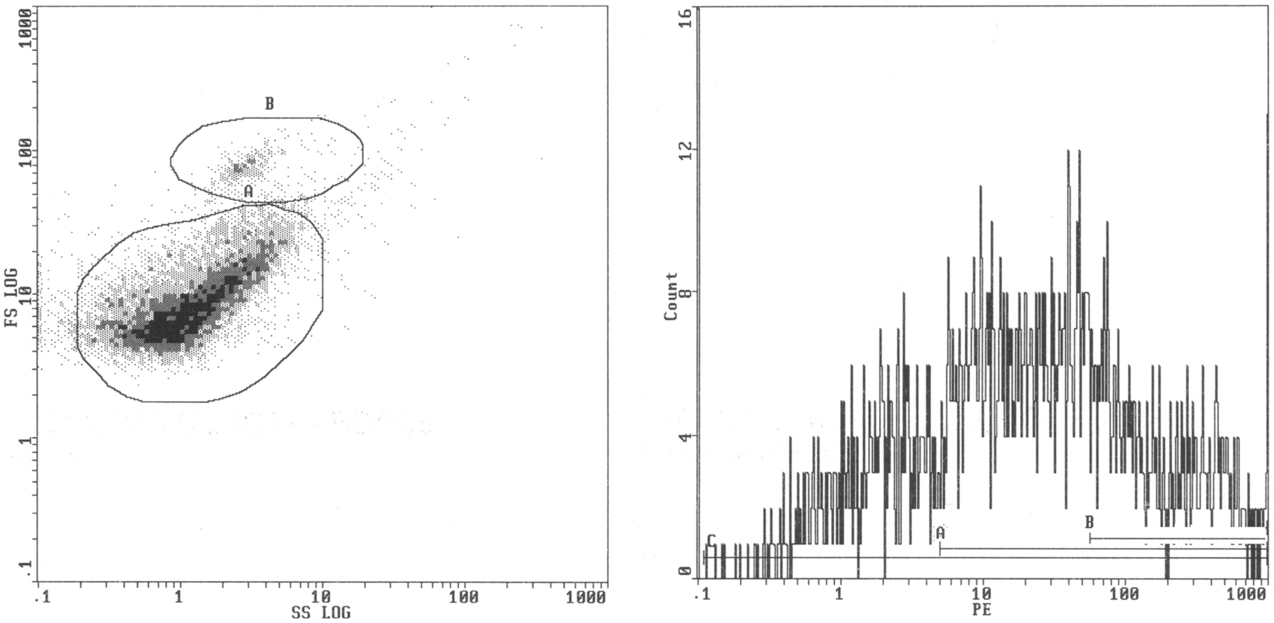

Supplementary figure 1.

MK obtained from stem cells were cultured and induced to differentiate for up to 14 days. At day 12 of differentiation cells were centrifuged at 1400 rpm for 10 min. at RT in order to separate progenitors from platelets. Supernatants were centrifuged for 20 min at 14000 rpm in order to pellet platelets and/or pro-platelets. These were stained by direct immunofluorescence with phycoerythrin (PE) conjugated CD61 (Biolegend) diluted 1:100, for 15 min at RT in the dark.

Acquisition was attained in a flow cytometer (Epics XL –MCL Coulter), provided with a 15 mV argon laser tuned at 488nm. Calibration of the instrument was performed with human platelet rich plasma (PRP). The upper size limit of events was defined in a logarithmic FS-SS dot plot using PRP.

Two gates were drawn: **A** (platelets): 82,9% of events and **B** (pro-platelets): 8.11 % (left panel in figure). Right panel shows PE-CD61 positive events **A:** 66.9 % and **B**: 80.9 % . Representative experiment.
