# Supplementary material for: From Human Megakaryocytes to Platelets: Effects of Aspirin on High-Mobility Group Box 1/Receptor for Advanced Glycation End Products Axis
Source: Front Immunol. 2018 Jan 12;8:1946. doi: 10.3389/fimmu.2017.01946 (PMC5770369; doi:10.3389/fimmu.2017.01946)
Supplement: Supplementary file 2 [file Image_2.PDF]

Supplementary figure 2

A

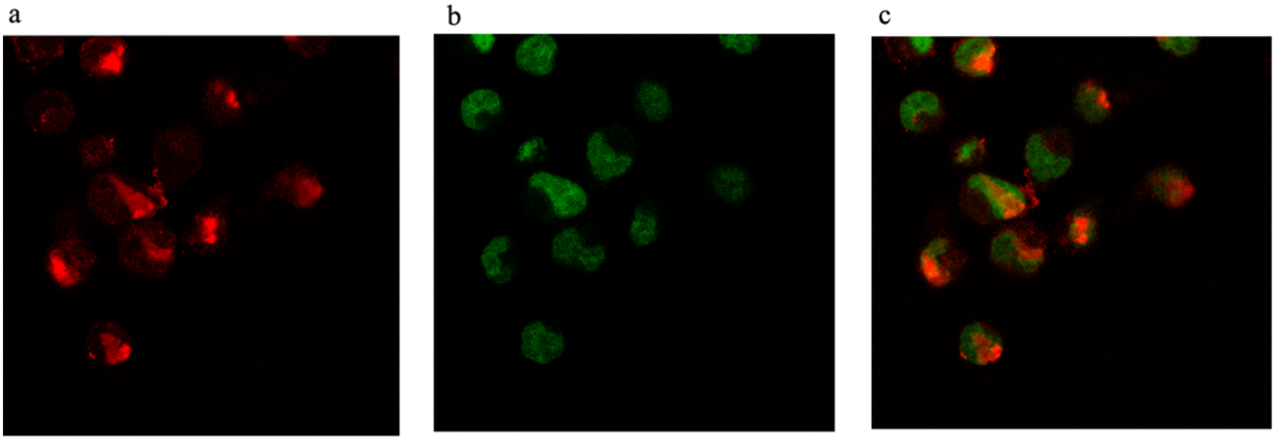

MKs day 7: a. HMGB1 red; b. Sytox green nuclei; c. merge (magnification 60X)

B

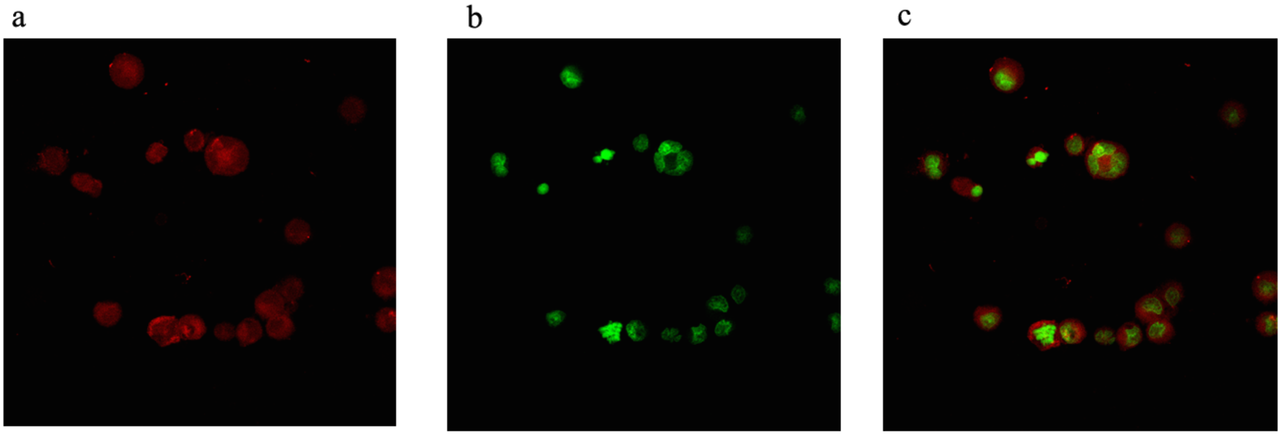

MKs day 14: a. HMGB1 red; b. Sytox green nuclei; c. merge (magnification 60X)

MKs obtained from HPCs were cultured and induced to differentiate. At day 7 (upper panel - A) and at day 14 (lower panel-B) cells were spotted on a glass slide, fixed with 4% paraformaldehyde in PBS for 30 min, washed with 0.1 M glycine for 20 min and permeabilized in 0.1% Triton X-100 for an additional 5 min. Primary antibodies (anti-HMGB1) were diluted according to manufacturer's instructions and added on spots for 45 minutes, then slides were washed and secondary antibodies (PE labeled anti IgG) were added for 30 min. At the end slides were washed with PBS, stained with Sytox green dye (100nM for 10 min), mounted with coverslips and kept at 4° C until imaging with confocal microscopy (Zeiss LSM-510).
